# Using the Age-Friendly 4Ms Framework to Redesign a Community Clinical Experience

**DOI:** 10.1093/geroni/igaa057.028

**Published:** 2020-12-16

**Authors:** Margaret Avallone, Staci Pacetti

**Affiliations:** Rutgers University-Camden, Camden, New Jersey, United States

## Abstract

The Age-Friendly 4Ms Framework (IHI/Hartford Foundation) was used to redesign an existing undergraduate nursing community experience, teaching students how to evaluate what matters to the individual, medications, mentation, and mobility. As part of the NJ Geriatric Workforce Enhancement Program (NJGWEP), a 5-year grant supported by DHHS-HRSA, ten senior nursing students joined a team of bilingual social workers, APNs, and a PharmD in an affordable housing urban highrise. This paper will describe the implementation and evaluation of a redesigned clinical experience using the 4Ms framework. Students visited older residents with bilingual social workers, performed health assessments and developed person-centered plans of care. Students presented resident cases during weekly interprofessional conferences to promote team collaboration and planning. Residents who screened positive for dementia were referred to an interprofessional Memory Assessment Program. Medications were reviewed using the Beers criteria, reconciled, and referred to primary care providers if appropriate. Fall risk was assessed and managed using the STEADI toolkit (CDC). Students were evaluated on attainment of geriatric-specific competencies, including medication review, cognition and depression screening, and fall risk assessments, by direct observations and interview. Following the semester-long experience, students completed a retrospective pretest/posttest survey to evaluate achievement of objectives based on the 4Ms. Mean scores for achievement of learning objectives ranged from 4.7-4.9 on a Likert scale of 1-5. Students identified barriers that older diverse individuals face when managing chronic health problems in the community. Students also valued the partnership with the social workers, stating, “We learned from each other.”

